# Knowledge, attitudes and practices regarding HIV/AIDS among senior high school students in Sekondi-Takoradi metropolis, Ghana

**DOI:** 10.4102/phcfm.v11i1.1875

**Published:** 2019-05-09

**Authors:** Seraphine M. Dzah, Elvis E. Tarkang, Prosper M. Lutala

**Affiliations:** 1 Department of Population and Behavioural Sciences, School of Public Health, University of Health and Allied Sciences, Ho, Ghana; 2 HIV/AIDS Prevention Research Network, Kumba, Cameroon; 3 School of Public Health and Family Medicine, College of Medicine, University of Malawi, Blantyre, Malawi

**Keywords:** knowledge, attitudes and practice, HIV/AIDS, senior high school students, Sekondi-Takoradi, Ghana

## Abstract

**Background:**

In Ghana, youths aged 15–24 years constitute the group most vulnerable to HIV infection. Inadequate knowledge, negative attitudes and risky practices are major hindrances to preventing the spread of HIV.

**Aim:**

This study sought to investigate the knowledge, attitudes and practices regarding HIV/AIDS among senior high school (SHS) students.

**Setting:**

Sekondi-Takoradi metropolis, Ghana.

**Methods:**

A descriptive, cross-sectional design was adopted, using a validated self-administered questionnaire, to collect data from a stratified sample of 294 senior students selected from three participating high schools in August 2017. The data collected were analysed using Stata version 12. Descriptive and inferential statistics were at a significance level of 0.05.

**Results:**

Among the participants, 61.6% had good knowledge about HIV/AIDS, 172 (58.5%) showed positive attitudes towards people living with HIV (PLHIV) and 79.1% reported HIV-related risky practices. We found a significant association between age and attitudes (*p* < 0.05). Poor knowledge was associated with being Muslim (aOR = 1.51 and 1.93; CI 1.19–1.91; *p* = 0.00) and being a student from school ‘F’ senior high school (F SHS) (aOR = 1.93; CI 1.71–2.18; *p* = 0.00). Bad attitude towards PLHIV and HIV was associated with ages 15–19 years (aOR = 3.20[2.58–3.96]; *p* = 0.03) *p* confirmed; and single marital status (aOR = 1.79[1.44–2.23]; *p* = 0.00). Bad practices were associated with ages 15–19 years (aOR = 1.72[1.41–2.11]; *p* = 0.08), belonging to the Akans ethnic group (aOR = 1.57[1.26–1.97]; *p* = 0.00) or being single (aOR = 1.79[1.44–2.23]; *p* = 0.00). Associations between misconceptions and HIV transmission were found: HIV can be transmitted by a handshake (aOR = 3.45[2.34–5.68]; *p* = 0.000), HIV can be cured (aOR = 2.01[2.12–5.04]; *p* = 0.004) and HIV/AIDS can be transmitted by witchcraft (aOR = 3.12[3.21–7.26]; *p* = 0.001).

**Conclusion:**

Participants generally had inadequate knowledge regarding HIV/AIDS, manifested negative attitudes towards PLHIV and also engaged in risky practices that might predispose them to HIV transmission. Our findings underscore the need for culturally adapted and age-oriented basic HIV information for youths in the metropolis on misconceptions about HIV transmission, negative attitudes of students towards PLHIV as well as the risky practices of students regarding HIV.

## Introduction

Human immunodeficiency virus/acquired immunodeficiency syndrome (HIV/AIDS) has rapidly spread to many countries over the years since 1981 and is becoming a global health challenge.^1^

Sub-Saharan Africa (SSA) is the worst affected region in the world, with about two-thirds of the afflicted people worldwide living here.^2^ According to UNICEF (2016) and UNAIDS (2018), the majority (about 80%) of the 1.8 million adolescents living with HIV live in SSA.^2,3^ Even in the general population, the majority (71%) of the people living with HIV (PLHIV) as well as new HIV infections (70%) and AIDS-related deaths (74%) worldwide are recorded in SSA.^4^ Human immunodeficiency virus/acquired immunodeficiency syndrome (HIV/AIDS) has become the leading cause of death in Africa, and it is responsible for one in every five deaths in SSA.^5^

Ghana registered 250 232 cases of PLHIV between 2006 and 2014.^6^ Of these, 92% were adults (15–49 years for women and 15–59 years for men) and 8% were children (6–59 months).^7^ The adult HIV incidence is estimated at 0.07%, with 11 356 new infections and 9248 AIDS-related deaths recorded. The prevalence of HIV in Ghana is described as generalised over the years, with a prevalence rate of more than 1% in the general population.^8^ The HIV prevalence in the western region of Ghana stands at 2.5%.^8^ Although the prevalence rates of HIV/AIDS in Ghana are not as high as in other African countries, the disease still poses a challenge to the country’s overall socio-economic development.^9^

Young adults, particularly those aged 15–24 years, are the group most vulnerable to HIV infection.^10,11^ This may be attributable to their engagement in risky life practices owing to lack of adequate information.^12^ Likewise, Ghanaians engage in their first sexual intercourse when they are in high school or of high school age.^1^ A study conducted in the Ashanti region of Ghana emphasised that most youth have pre-marital sex when young.^13^ Furthermore, youth present specific challenges that predispose them to HIV, some of them being lack of correct health information, lack of access to adequate reproductive health services, economic exploitation, changing lifestyles, global conflicts,^14^ exchange of sex to meet their needs and substance use.^15^

Knowledge, attitudes and practices (KAPs) regarding HIV/AIDS serve as the cornerstones in the fight against HIV. Adequate knowledge regarding HIV/AIDS is a powerful way of promoting positive attitudes as well as engaging in safe practices.^16^ The attitude regarding HIV/AIDS in turn is expected to determine people’s sexual behaviour.^16^ Many prevention programmes have focussed on increasing knowledge on transmission, with the aim of overcoming misconceptions that could prevent behavioural change towards safe practices and also reduce the stigma against PLHIV.^16^

Several studies have been conducted in Africa and beyond to investigate KAP levels among students. These studies found that the knowledge of students regarding HIV was either average or poor, with misconceptions on high-risk practices among participants and a negative attitude towards PLHIV. Misconceptions were equally found in most KAP studies conducted among youths in different parts of Africa (Nigeria, Botswana, Gabon and other African countries) and beyond.^1,17^

A study conducted in Nigeria to identify knowledge of HIV infection among secondary school students in Port Harcourt found that only 7.1% of participants listed the four modes of transmission, namely sexual intercourse, blood transfusion, mother to child (vertical) transmission and intravenous drug use. The above four modes of transmission were identified by only 31%, 14.4%, 9.1% and 8%, respectively. Only 0.7% identified all the preventive measures.^18^ Another survey in western Nigeria assessing the level of awareness, knowledge and attitude towards HIV/AIDS among secondary school students in Atisbo Local Government Area, Nigeria, showed that participants possess relatively good knowledge of HIV/AIDS, reasonable knowledge of safe sexual practices and positive attitude towards sexuality, HIV/AIDS and people living with PLHIV.^5^ But another study in Gabon which assessed HIV-related KAPs of high school and college students showed that students have inadequate information about HIV/AIDS transmission and prevention. Half of the respondents were aware of HIV transmission through sexual intercourse (55.7%), from mother to child (48.3%) and through sharing needles or syringes (51.8%), and 25% used condoms despite 15% being aware of unsafe practices with regard to HIV transmission.^19^

Misconceptions were also found in studies conducted in India^20^ and Afghanistan^21^ where participants believed, for example, that HIV can be transmitted through toilet seats^21^ or mosquito bites.^20,21^ Negative attitude towards PLHIV was also reported by another study.^20^

Good knowledge of HIV does not always translate into good behaviour and/or safe practices. In Botswana, for example, a study conducted previously showed that half of the students could be perceived to be at risk of HIV, while the same participants thought that each sexually active student should be aware of his HIV status through regular testing.^22^

Youths need information to make responsible choices concerning their sexual behaviour. However, there is scarcity of studies addressing KAPs in HIV/AIDS among high school students in Ghana.

Although HIV/AIDS-related KAPs have been reported in studies from other countries and towns in Ghana, to our knowledge, there was no such information for students in Sekondi-Takoradi metropolis, Ghana.

The current study was therefore conducted to find out the KAPs regarding HIV/AIDS among senior high school (SHS) students in the Sekondi-Takoradi metropolis. This could serve as a guide to providing relevant information to health policymakers and other stakeholders in developing strategies related to supporting adolescents in improving their knowledge, attitudes, practices, values and skills needed to achieve the requisite behavioural changes to protect them from HIV infection in the Secondi-Takoradi metropolis of Ghana.

## Methods

### Study design

A cross-sectional, descriptive study design was adopted with quantitative data collection methods. The choice of this study design was to obtain information which is a snapshot of a population at a certain time, allowing conclusions about phenomena to be drawn across a wide population.^19^

### Study site description

Sekondi-Takoradi metropolis is located in the south-eastern part of the western region of Ghana. The metropolis is bordered to the west by Ahanta West District and to the east by Shama District. To the south of the metropolis is the Atlantic Ocean and to the north is the Wassa East District. It covers a land size of 191.7 km^2^, and Sekondi-Takoradi is the regional administrative capital. Although the smallest district in terms of land size, the Sekondi-Takoradi metropolis is the most urbanised among the 22 districts in the region. The population of Sekondi-Takoradi metropolis, according to the 2010 Population and Housing Census (PHC), was 559 548 inhabitants, accounting for 23.5% of the region’s total population.^23^

### Study population and sampling strategy

The study population included students (both males and females) from three selected SHSs in the Sekondi-Takoradi metropolis – all boarding schools. This population was used because most of the participants (students) were aged between 15 and 24 years, which coincide with the age group most vulnerable to HIV/AIDS infection.^12^ All students who were in SHS and were present and consented to participate were included. The minimum sample size was obtained for this study by using the Cochran formula.^24^

$$n=\frac{Z^{2}pq}{d^{2}},$$ [Eqn 1]

where,

*n* = sample size,

Z = Z_score_,

*p* = estimated proportion of an attribute that is present in the population,

*q* = 1-*p*,

*d* = margin of error.

It was based on the assumption of a margin of error of 0.03, 95% confidence level and an estimated proportion of HIV knowledge of Ghanaian youth (15–24 years) as 75.4% ^25^ and 0.03 (3%) non-response rate.

$$\begin{array}{c} \text{A sample size }(n)=\frac{{\left(1.92\right)}^{2}\times 0.754\times (1-0.754)}{{\left(0.05\right)}^{2}}\\ n=285 \end{array}$$ [Eqn 2]

Adding a non-response rate of 3%, *n* = (285*0.03) + 285 = 294. Therefore, a total of 294 students who met the study inclusion criteria were recruited to this study.

A stratified sampling technique was employed in the study, where a proportional number of the students were drawn from each form (one and two) level. Calculation of the number of respondents in each grade level was based on the proportion of the population in the school register (sampling frame). That is, the study sample size divided by the total number in the school multiplied by the total number of students in each grade level. Then, using the sample size determined for each form, the number of respondents per form (grade level) was selected using simple random sampling. This was then computed to achieve the total sample size of 294.

#### Data collection procedure

A standard set of questionnaires was distributed among the students. Before administering the questionnaire, the nature of the study was explained to the students. They were assured of anonymity and confidentiality of their responses. The face and content validities of the instrument were ensured by comparing items with previous similar studies and by matching them with the stated objectives. In addition, a copy of the prepared questionnaire was made available to the project supervisor for vetting, review and careful scrutiny for the necessary amendments and corrections. Also, a pretest was carried out among 20 students in two schools that were not part of the study to ensure the reliability of the instrument. The principal investigator trained three data collectors about informed consent and the different sections of the questionnaires. The questionnaires were administered by the principal investigator and the three trained data collectors to all qualified students in the study. The questionnaires were administered during regular school hours. Students who volunteered to participate were made to sit apart and asked not to communicate with each other during the administration of the questionnaires so as to encourage honest responses, while the teacher was outside the classroom. After collecting the completed questionnaires, students were thanked for their participation in the study.

#### Data analysis

The data collected were entered into Epi Data Entry version 3.1 and exported to Stata version 12.0 for cleaning and analyses. Descriptive statistics such as frequencies, percentages, tables and charts were used to present the data. Logistic regression was also used to test associations between socio-demographic variables and KAPs of respondents regarding HIV/AIDS at the significance level of 0.05.

### Ethical considerations

Ethical approval for the study was sought from the Ghana Health Service Ethics Review Committee (GHS-ERC: 86/05/2017) with the help of the School of Public Health, University of Health and Allied Sciences, Ho, Ghana. Moreover, local permission and approval for the study was obtained from the headmasters of the various schools where the study was conducted.

## Results

### Socio-demographic characteristics of the respondents

The study comprised students aged 15–20 years. It was conducted in three schools in the Sekondi-Takoradi metropolis. (The letters S, F and T were used as pseudonyms for the schools to ensure anonymity.)

Table 1 shows that the majority of the respondents were females (164; 55.8%) and aged between 15 and 17 years (244; 83.0%). The mean age of the respondents was approximately 17.0 with a standard deviation of ±0.981. Respondents were distributed almost equally across three different schools. Participants were mostly Christians (278; 94.6%), and from the Fante ethnic group (103; 35.0%). A vast majority of the respondents was single at the time of the study (284; 96.6%), had fathers who went to college (71; 24.2%), and had an education level of 2 years (200; 68%).

**TABLE 1:** Demographic distribution of the respondents.

Demography	Frequency^†^	Per cent
**Gender**		
Male	164	55.8
Female	130	44.2
**Age group**		
15–17 years	244	83.0
18–20 years	50	17.0
**Religion**		
Christian	278	94.6
Islam	15	5.1
African Indigenous	1	0.3
**Father’s educational status**		
College	78	26.5
University	71	24.2
O-Level	59	20.1
Senior High School	43	14.6
Junior High School	30	10.2
Primary	13	4.4
**Ethnic group**		
Fante	103	35.0
Akan	84	28.6
Ewe	39	13.3
Ahanta	28	9.5
Nzema	28	9.5
Other	12	4.1
**Marital status**		
Single	284	96.6
Dating	9	3.1
Married	1	0.3
**Education/Grade level**		
Two	200	68.0
One	94	32.0

Note: O-level is the abbreviation of ordinary level. It is one of the two parts of the general certificate of education (GCE). It is the final certificate for secondary school, to be taken at fifth form or year 11 at approximately age 17.

† *n* = 294.

### Knowledge of respondents regarding HIV/AIDS

**FIGURE 1:**
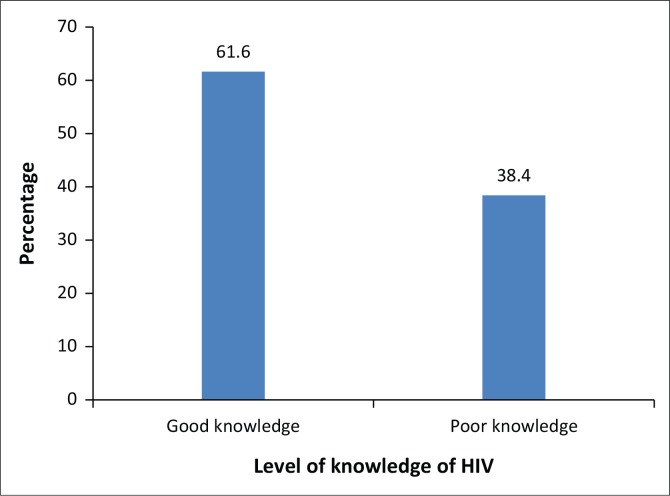
Level of knowledge of HIV/AIDS.

From Table 2, it can be seen that the majority of the respondents (286; 97.3%) knew that HIV/AIDS can be transmitted via sexual intercourse, from mother to child (252; 85.7%), through sharing needles or syringes (274; 93.2%) and through blood transfusion (278; 94.6%). Most respondents knew that HIV/AIDS cannot be transmitted through handshake (239; 81.3%), by sharing clothes with an HIV-infected person (205; 69.7%) and by mosquito bite (206; 70.1%). The majority of the respondents (217; 73.8%) knew HIV/AIDS cannot be transmitted by witchcraft, while a slight majority (178; 60.5%) knew it cannot be transmitted by using the same toilet seat as an HIV-positive patient. Notably, only a slight majority (174; 59.2%) knew HIV/AIDS is not curable. The human immunodeficiency virus transmission is associated with the following misconceptions: HIV can be transmitted by handshake (OR = 3.45; 95% CI 2.34–5.68; *p* = 0.000) and by witchcraft (OR = 3.12; 95% CI 3.21–7.26; *p* = 0.001). However, from Figure 1, it is clear that only a slight majority of the respondents had good knowledge regarding HIV/AIDS (61.6%).

**TABLE 2:** Knowledge of respondents regarding HIV/AIDS.

Variable	Frequency^†^	AOR	95% CI	*p*
**HIV can be transmitted by sexual intercourse**				
Yes	286	Ref.	-	-
No	5	1.2	1.94–4.07	0.062
Do not know	3	1.3	0.45–3.12	0.321
**HIV can be transmitted from mother to child**				
Yes	252	Ref.	-	-
No	29	1.89	0.12–6.67	0.435
Do not know	13	0.78	0.21–2.87	0.671
**HIV can be transmitted by sharing needle or syringe**				
Yes	274	Ref.	-	-
No	15	1.89	0.043–1.976	0.073
Do not know	5	0.19	0.90–1.82	0.831
**HIV can be transmitted by blood transfusion**				
Yes	278	Ref.	-	-
No	9	0.13	0.09–4.54	0.654
Do not know	7	0.9	0.65–2.81	0.328
**HIV can be transmitted by handshake**				
Yes	39	3.45	2.34–5.68	0.000
No	239	Ref.	-	-
Do not know	16	2.05	0.38–1.97	0.981
**HIV can be transmitted by wearing the clothes of an HIV-positive person**				
Yes	50	1.23	0.94–2.13	0.076
No	205	Ref.	-	-
Do not know	39	0.21	0.72–3.10	0.234
**HIV/AIDS can be transmitted by mosquito bite**				
Yes	58	2.13	0.54–2.98	0.561
No	206	Ref.	-	-
Do not know	30	0.31	0.98–2.10	0.391
**HIV/AIDS can be cured**				
Yes	82	2.01	2.12–5.04	0.004
No	174	Ref.	-	-
Do not know	38	0.56	0.81–2.09	0.213
**HIV/AIDS can be transmitted by witchcraft**				
Yes	44	3.12	3.21–7.26	0.001
No	217	Ref.	-	-
Do not know	33	0.98	0.17–1.30	0.912
**HIV/AIDS can be transmitted by using the same toilet seat as an HIV-positive person**				
Yes	69	1.42	0.16–1.19	0.813
No	178	Ref.	-	-
Do not know	47	2.9	0.27–4.06	0.67

AOR, adjusted odds ratio; CI, confidence interval; Ref., reference class.

† *n* = 294.

### Attitudes of respondents regarding HIV/AIDS

It could be noted from Table 3 that the majority of the respondents (233; 79.2%) were willing to care for their HIV-positive relatives. However, 169 (57.5%) said they would not eat from the same bowl used by the PLHIV. However, the majority of the respondents (219; 74.5%) were willing to continue their friendship with their HIV-positive friends. On the contrary, most of them, (201; 68.4%) said they would not buy items from shopkeepers with HIV/AIDS. Most of the respondents said that students (228; 77.6%) and teachers (224; 76.2%) who are HIV positive should be allowed to continue their studies and teaching, respectively. The majority (223; 75.9%) said they had never refused to take care of HIV/AIDS patients.

**TABLE 3:** Attitudes of respondents regarding HIV/AIDS.

Variable	Yes		No		Do not know	
		
	*N*	%	*N*	%	*N*	%
Would you be willing to care for your relative with HIV/AIDS?	233	79.2	57	19.4	4	1.4
Would you eat from the same bowl used by a person living with HIV?	124	42.2	169	57.5	1	0.3
Would you drink from the same cup used by a person living with HIV?	81	27.6	212	72.1	1	0.3
If your friend is HIV-positive, would you continue your friendship with him/her?	219	74.5	74	25.2	1	0.3
If a shopkeeper or food seller is HIV-positive, would you buy items from him/her?	92	31.3	201	68.4	1	0.3
If a student is HIV-positive, should she/he be allowed to continue his/her studying in school?	228	77.6	66	22.5	0	-
If a teacher is HIV-positive, should she/he be allowed to continue his/her teaching in school?	224	76.2	70	23.8	0	-
Have you ever refused to care for a person with HIV/AIDS?	71	24.1	223	75.9	0	-

### Excluded in the analysis

From Table 5, it is clear that Muslims (OR = 1.6; CI 1.23–2.06; *p* = 0.00) on one side, and Fante (OR = 1.74; CI 1.36–2.22; *p* =** 0.05) and Ewe (OR = 1.54; CI 1.17–2.02; *p* = 0.02) ethnic groups on the other side, were almost two times more likely to have poor HIV/AIDS knowledge than Christians and others ethnic groups.

**TABLE 4:** Practices of respondents regarding HIV/AIDS.

Question	Frequency	Per cent
**Had sex before?**		
Yes	77	26.2
No	217	73.8
Total	**274**	**100.0**
**Used condom during first sexual intercourse? (***n* **= 77)**		
Yes	37	48.1
No	40	51.9
Total	77	100.0
**Used condom during last sexual intercourse?**		
Yes	39	50.6
No	38	49.4
**How regularly do you use condom during sexual intercourse?**		
Sometimes	15	19.5
Always	32	41.5
Not at all	30	39.0
**Have you had multiple sex partners in the past 1 year?**		
Yes	42	54.5
No	21	27.3
Do not know	14	18.2
**Do you have a sex partner at present?**		
Yes	49	63.6
No	28	36.4
**Do you use injection drug equipment? (***n* **= 280)**		
Yes	18	6.4
No	262	93.6
**Do you share equipment with other drug users? (***n* **= 18)**		
Yes	14	77.8
No	4	22.2
**Have you done an HIV test before? (***n* **= 294)**		
Yes	16	5.4
No	278	94.6

**TABLE 5:** Comparison of levels of knowledge of HIV/AIDS among students aged 15–24 years by socio-demographic characteristics with 95% confidence.

Demographic variable	Knowledge (Poor = 113 )		COR	95% CI	*p*	AOR	95% CI	*p*

	*n*	%						
**Age**								
15–19 years	94	83.20	0.28	1.00–1.63	0.23	0.2	0.74–0.92	0.14
20–24 years	19	16.80	Ref.	-	-	Ref.	-	-
**Sex**								
Male	50	44.30	Ref.	-	-	Ref.	-	-
Female	63	55.70	1.13	0.92–1.38	0.24	1.09	0.92–1.29	0.32
**Religion**								
Christian	105	92.90	Ref.	-	-	Ref.	-	-
Islam	7	6.20	1.6	1.23–2.06	0.00	1.51	1.19–1.91	0.00
African Indigenous	1	0.90	0.81	0.62–1.06	0.13	0.81	0.63–1.05	0.11
**Name of school**								
F SHS	37	32.70	1.3	1.11–1.52	0.01	1.93	1.71–2.18	0.06
S College	36	31.90	Ref.	-	-	Ref.	-	-
T SHS	40	35.40	0.77	0.56–1.05	0.10	0.46	0.35–0.62	0.00
**Ethnic group**								
Ahanta	10	8.90	1.34	1.15–1.58	0.00	2.84	2.55–3.18	0.00
Nzema	14	12.40	Ref.	-	-	Ref.	-	-
Fante	34	30.10	1.74	1.36–2.22	0.05	1.57	1.26–1.97	0.03
Akan	29	25.70	0.48	0.34–0.67	0.00	0.38	0.28–0.50	0.00
Ewe	20	17.60	1.54	1.17–2.02	0.02	1.24	0.98–1.57	0.00
Other	6	3.30	1.43	1.08–1.91	0.01	1.14	0.89–1.47	0.31
**Marital status**								
Married	0		Ref.	-	-	Ref.	-	-
Single	110	97.40	1.27	1.01–1.59	0.04	1.45	1.30–1.63	0.00
Dating	3	2.60	1.07	0.78–1.45	0.69	0.89	0.75–1.08	0.25
**Form**								
One	43	38.10	0.75	0.49–1.15	0.19	0.76	0.51–1.15	0.20
Two	70	61.90	0.51	0.33–0.77	0.01	0.64	0.43–0.95	0.03

SHS, senior high school; Ref., reference class; COR, crude odds ratio; AOR, adjusted odds ratio; CI, confidence interval.

On multivariate analysis (Table 5), the Muslims and students from F SHS were almost two (aOR = 1.51 and 1.93; CI 1.19–1.91 and 1.71–2.18; *p* = 0.00 each) times more likely to have poor knowledge, while the Ahantas were three times more likely to have poor knowledge compared to the Nzemas. All these associations were statistically significant after adjusting for covariants.

**FIGURE 2:**
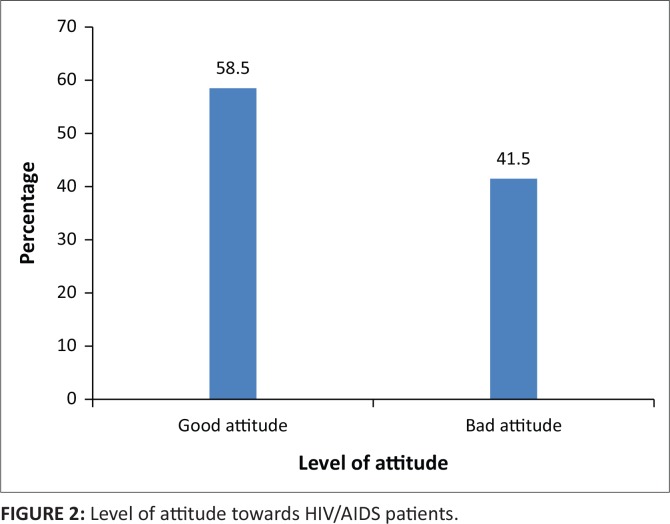
Level of attitude towards HIV/AIDS patients.

Generally, a slight majority of the respondents (58.5%) had positive attitudes towards PLHIV (see Figure 2).

### Practices of respondents regarding HIV/AIDS

Table 4 presents the results of the practices of respondents regarding HIV/AIDS. Most of the respondents (217; 73.8%) said they had never had sex before. The majority (41; 51.9%) of those who had experienced sex before did not use a condom during their first sexual encounter. However, the majority (39; 50.6%) used a condom during their last sexual encounter. Only 32 (41.5%) of the sexually active respondents always used a condom during sexual encounters. The findings also showed that more than half (42; 54.5%) of the sexually active respondents had multiple sex partners in the year before this study. During the current study, the majority of the sexually active respondents (49; 63.6%) had a sex partner. The study findings indicated that the majority of the respondents (262; 3.6%) was not using injection drugs. The majority (14; 77.8%) of those who used injection drug equipment said they shared with other users. The majority of the respondents (278; 94.6%) said they had never had an HIV test before.

**FIGURE 3:**
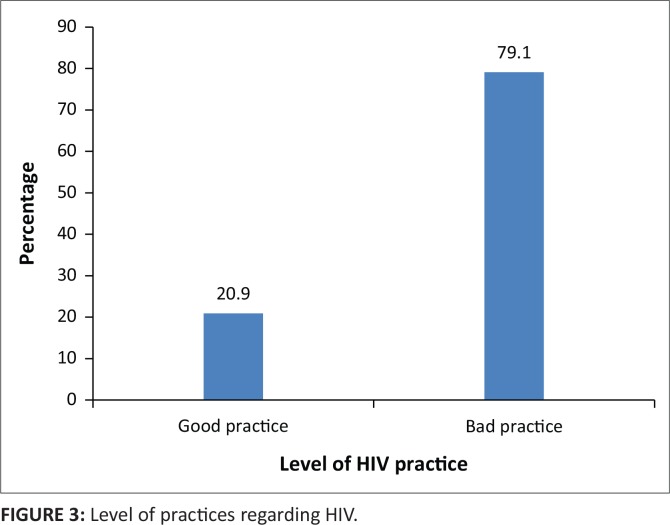
Level of practices regarding HIV.

The results revealed that the majority of the respondents (79.1%) was engaging in bad practices regarding HIV (see Figure 3).

### Factors associated with knowledge, attitudes and practices regarding HIV/AIDS

From Table 6, it is clear that students who were aged 15–19 years were almost two times (aOR = 1.72 [1.41–2.11]; *p* = 0.00) more likely to have bad practice towards HIV/AIDS than those who were aged 20–24 years, while the Akans and those who belong to other ethnic groups and those who were not married were all almost two times more likely to have bad practices regarding HIV/AIDS. On multivariate analysis, those students aged 15–19 years, Akans and singles were almost two times more likely to have bad practice.

**TABLE 6:** Comparison of prevention practice regarding HIV/AIDS among students aged 15–24 years by socio-demographic characteristics with 95% confidence.

Demographic variable	Practice (Bad = 53)		COR	95% CI	*p*	AOR	95% CI	*p*

	*n*	%						
**Age**								
15–19 years	41	77.4	2.03	1.59–2.61	0.00	1.72	1.41–2.11	0.08
20–24 years	12	22.6	Ref.	-	-	Ref.	-	-
**Sex**								
Male	28	52.8	Ref.	-	-	Ref.	-	-
Female	25	47.2	0.51	0.33–0.77	0.00	0.64	0.43–0.95	0.03
**Religion**								
Christian	51	96.2	Ref.	-	-	Ref.	-	-
Islam	2	3.8	0.57	0.49–0.69	0.04	0.75	0.65–0.85	0.06
African Indigenous	0	0.0	0.78	0.67–0.91	0.00	0.89	0.77–1.02	0.09
**Ethnic group**								
Ahanta	4	7.6	0.92	0.78–1.07	0.28	1.23	1.07–1.42	0.00
Nzema	5	9.4	Ref.	-	-	Ref.	-	-
Fante	18	34.0	1.17	0.92–1.48	0.19	1.17	0.93–1.47	0.18
Akan	19	35.8	1.74	1.36–2.22	0.00	1.57	1.26–1.97	0.00
Ewe	6	11.3	0.48	0.34–0.67	0.00	0.38	0.28–0.50	0.00
Other	1	1.9	1.54	1.17–2.02	0.00	1.24	0.98–1.57	0.07
**Marital status**								
Married	0	0.0	Ref.	-	-	Ref.	-	-
Single	50	94.0	1.90	1.53–2.23	0.00	1.79	1.44–2.23	0.00
Dating	3	-	1.16	0.90–1.49	0.25	1.13	0.89–1.43	0.31
**Form**								
One	16	30.0	1.11	0.85–1.45	0.04	0.74	0.63–0.88	0.06
Two	37	70.0	1.17	0.92–1.47	0.20	1.04	0.47–1.25	0.68

Ref., reference class; COR, crude odds ratio; AOR, adjusted odds ratio; CI, confidence interval; *p*, probability at 0.05%; Form, level of secondary school.

Table 7 shows that students aged 15–19 years were four times (OR = 4.36; CI 3.24–5.86; *p* = 0.00) more likely to have a bad attitude towards HIV/AIDS than those who were aged 20–24 years, while the Fantes were almost three times more likely to have a bad attitude towards PLHIV than the Nzemas. On multivariate analysis, those students who were aged 15–19 years were three times more likely to have a bad attitude towards PLHIV than those aged 20–24 years (aOR = 3.20; CI 2.58–3.96; *p* = 0.03).

**TABLE 7:** Socio-demographic correlates of students’ attitude towards HIV/AIDS.

Demographic variables	Attitudes							

	Bad (53)		COR			AOR		
		
	*n*	%	*n*	95% CI	*p*	*n*	95% CI	*p*
**Age**								
15–19 years	41	77.4	4.36	3.24–5.86	0.000	3.2	2.58–3.96	0.03
20–24 years	12	22.6	Ref.	-	-	Ref.	-	-
**Sex**								
Male	28	52.8	Ref.			Ref.		
Female	25	47.2	0.51	0.33–0.77	0.000	0.64	0.43–0.95	0.03
**Religion**								
Christian	51	96.2	Ref.	-	-	Ref.	-	-
Islam	2	3.8	0.57	0.42–0.77	0.000	0.34	0.27–0.45	0.00
African Indigenous	0	0.0	1.49	1.08–2.06	0.020	1.01	0.75–1.36	0.95
**Ethnic group**								
Ahanta	4	7.6	1.03	0.76–1.39	0.870	0.37	0.29–0.48	0.00
Nzema	5	9.4	Ref.	-	-	Ref.	-	-
Fante	18	34.0	2.72	1.92–3.85	0.000	1.3	0.95–1.78	0.10
Akan	19	35.8	1.14	0.83–1.57	0.410	0.62	0.47–0.82	0.00
Ewe	6	11.3	0.86	0.63–1.18	0.358	0.38	0.29–0.50	0.03
Other	1	1.9	0.57	0.42–0.77	0.000	0.34	0.27–0.45	0.02
**Marital status**								
Married	0	0.000	Ref.	-	-	Ref.	-	-
Single	50	94.3	1.93	1.53–2.43	0.000	1.79	1.44–2.23	0.00
Dating	3	5.7	1.16	0.90–1.49	0.250	1.13	0.89–1.43	0.31
**Form**								
One	16	30.2	1.11	0.85–1.45	0.460	0.74	0.63–0.88	0.00
Two	37	69.8	1.17	0.92–1.47	0.200	1.04	0.87–1.23	0.30

Ref., reference class; COR, crude odds ratio; AOR, adjusted odds ratio; CI, confidence interval; *p*, probability at 0.05%; Form, level of secondary school.

## Discussion

This study aimed at investigating KAPs regarding HIV/AIDS among SHS students in Ghana. In our KAP study, we found students were more knowledgeable about modes of transmission in general. The attitudes of participants towards HIV and AIDS patients were mixed. More than three-quarters of participants were engaged in bad risk practices. However, few misconceptions were found among participants: 69.7% knew that HIV cannot be transmitted by sharing clothes with an HIV-infected person, and 60.5% knew that HIV cannot be transmitted by using the same toilet seat as an HIV-positive person. Notably, only 174 (59.2%) knew that HIV/AIDS is not curable. Factors associated with poor knowledge of HIV/AIDS (Muslim religion, attending F and T SHSs (F and T senior high schools), belonging to the Ahanta ethnic group and being single), bad attitude towards HIV/AIDS (being aged between 15 and 19 years, being single and belonging to the Fante ethnic group) and bad practices (age between 15 and 19 years, belonging to the Akan ethnic group and being single) were also determined.

The current results indicated that the majority of the respondents knew that HIV could be transmitted by sexual intercourse, from mother to child, by sharing contaminated needles or syringes and through blood transfusion. These findings are in agreement with two Ghanaian studies.^10,26^ Agyemang et al.^10^ conducted a cross-sectional study among high school students in the Ejura-Sekyedumase district of Ghana and found that sexual intercourse was the most commonly identified means of HIV transmission (95.0%), followed by sharing of razors, needles or other sharp items with an infected person (86.0%) and blood transfusion (37.1%).^10^ Furthermore, Agyekum and Suapim,^26^ in a cross-sectional study among high school girls in Ghana, revealed that almost all the respondents (90.0%) were aware that HIV/AIDS could be transmitted through bodily fluids like blood, semen, vaginal secretions and breast milk.^26^ Exposure to several awareness campaigns partially can explain these results; however, our study showed an adjusted association between knowledge of HIV and Muslim religion, attending an FSHS, belonging to the Ahanta ethnic group and being single. This association with the lower age group is similar to what has been seen in Ghana, where only one out of two adolescents has a comprehensive knowledge of HIV.^19^ The low self-efficacy measures and the difficult access to protective measures such as condoms among youths owing to resistance and shame from selling points could as well explain this association.

Single marital status is associated with the odds of little knowledge and odds of getting infected, as demonstrated by Carlos in Congo, where being single, divorced or widowed increases the likelihood of getting infected almost ten times.^27^ The big difference in the odds of infection in single participants in Congo compared to ours could be explained by specific characteristics of participants. While our study was focussed on school-going youth, the Congolese study’s participants were a mix of youths and adults, with a median age of 34.9 years.^27^

There is a direct relation between sexual behaviour and misconceptions in HIV knowledge.^28^ Most of the respondents in the current study knew that HIV/AIDS cannot be transmitted through handshake (81.3%), by wearing the clothes of an HIV-infected person (69.7%), by mosquito bite (70.1%), through witchcraft (73.8%) or by using the same toilet seat as an HIV-positive person (60.5%). These findings contravene those of Christane et al.,^19^ who reported in their study conducted in Libreville, Gabon, using a cross-sectional survey, that nearly half of their respondents incorrectly thought that HIV could be transmitted by eating from the same plate, drinking from the same glass, wearing the same clothes and sharing a toilet with a PLHIV.^19^ These differences might have been a result of increased level of HIV education programmes rolled-out in the Sekondi-Takoradi metropolis compared to that in Libreville, Gabon. However, the finding regarding mosquitoes transmitting HIV agrees with theirs as 67.7% of their respondents correctly answered that mosquitoes do not transmit HIV compared to 70.1% in the current study. These findings also supported Tarkang, who conducted a quantitative, non-experimental, descriptive, explorative and correlational research in Kumba, Cameroon, and reported that respondents showed some misconception that HIV/AIDS can be transmitted through mosquito bite (38.4%), through toilet seats (21.8%) and through handshake with an infected person (5.3%) as compared to 19.5%, 23.5% and 13.3%, respectively, in this study.^29^ These similarities could be attributed to some degree of misconception that many people still have regarding HIV transmission across Ghana and even Africa. Just more than 40% of the respondents in the current study had the misconception that HIV is curable. The factors associated with these misconceptions in our study are taking care for a relative with HIV and sharing the same plate with an AIDS patient, which increases the risk by 2 and 3 times, respectively. These factors were explored in a Congolese study as well which showed an association between misconceptions and risk of transmission such as HIV can be transmitted by sorcery and by mosquito bite.^27^ The implication of this kind of misconception is that these respondents would indulge in practices that might decrease the support system for the patient in an incurable chronic disease where a strong social support system plays a critical role in the resilience of the patient or the HIV-positive individual. This will definitely impact on the overall management and the outcomes thereof. There is therefore a need to implement health promotion programmes among students in the study area in order to improve their knowledge level regarding HIV/AIDS. The same study conducted in Kinshasa, Congo (Democratic Republic of Congo [DRC]), reported more than three of these misconceptions (the thinking that an HIV-positive person cannot look healthy and that HIV is transmitted by sorcery; it is God’s punishment; and it spreads through a kiss on the mouth, mosquitoes, coughs/sneezes or undercooked food) were associated with HIV.^27^ However, even if these associations of misconceptions on HIV status were found in both the studies, our study uses a slightly different questionnaire to detect misconceptions, which limits the comparison between the two studies. Nonetheless, in the multivariable analysis, three misconceptions have shown an association with knowledge of transmission modes (HIV can be transmitted by wearing HIV-positive patient’s clothes, by handshake or by mosquito bite). Furthermore, like in the Congolese study,^27^ an association between misconceptions and socio-demographic variables was not established in our study. This decreases somewhat the ability of our results in designing interventions tailored to participants aimed at reducing the impact of those misconceptions on their HIV risk.

Overall, the knowledge level of respondents of HIV/AIDS was found to be encouraging as the majority (61.6%) had good knowledge regarding HIV/AIDS. This finding contradicts that of Agyemang et al.,^10^ who revealed in their study in the Ejura-Sekyedumase district of Ghana that 33.6% of their study respondents had good knowledge regarding HIV/AIDS, and that of Huda and Amanullah,^12^ who carried out a cross-sectional study in Bangladesh and demonstrated that 34.4% of the students had good knowledge regarding HIV/AIDS.^12^ The significant level of knowledge of respondents in this study testifies to the positive impact of the HIV educational programmes organised in the Sekondi-Takoradi Municipality by the Ghana AIDS Commission and other organisations over the years. However, knowledge regarding HIV/AIDS should be universal. Therefore, the accurate knowledge level of 61.6% reported in the current study should be considered inadequate. This calls for concerted efforts and health promotion programmes among SHS students in Secondi-Takoradi, to increase their level of knowledge regarding HIV/AIDS to 100%. Inaccurate knowledge might lead to negative attitudes towards PLHIV, which could in turn lead to stigma and discrimination.

Attitudes of respondents towards PLHIV showed that the majority (79.3%) of them was willing to care for their relatives with HIV/AIDS. However, 57.5% of them said they would not eat from the same bowl used by a PLHIV. A number of participants believed that they cannot share a cup with a PLHIV or buy from an HIV-positive shopkeeper, while more than three-quarters saw no harm in allowing teachers and students to continue with their jobs or studies while living with HIV.

These attitudes, despite being ambiguous on some points, are similar to the findings of Nubed and Akoachere,^1^ namely that 52.5% of students had positive attitudes towards PLHIV and 47.5% had negative attitudes, compared to 58.5% and 41.5% of this study, respectively.

Their cross-sectional study in Fako Division, Cameroon, revealed that 52.6% of respondents indicated their willingness to take care of a sick HIV-positive relative or continue friendship with an HIV-positive friend, while 56.9% could buy food and other goods from an HIV-positive person. Their study further revealed that the majority of the participants accepted that an HIV-positive student should be allowed to continue her/his studies (71.6%) and that an HIV-positive teacher should be allowed to continue her/his teaching profession (75.0%).^1^ The current findings differed from a cross-sectional study by Christane et al.,^19^ which found that less than half of the respondents showed positive attitudes on issues such as buying items from an HIV-positive shopkeeper or food seller, allowing an HIV-positive student to continue her/his studying in school and allowing an HIV-positive teacher to continue her/his teaching in school. However, the positive attitude among 58.5% of the respondents in the current study agrees with theirs as they reported in their study that 55.7% of their respondents manifested a positive attitude towards PLHIV. The current findings imply that the study respondents had some level of acceptance of HIV-positive persons into society in the Sekondi-Takoradi metropolis. It also means being HIV-positive is not an end to one’s career or profession.

However, the positive attitude among 58.5% of participants towards PLHIV reported in the current study falls short of the zero-discrimination against PLHIV as prescribed by UNAIDS.^30^ This could be because of the low level of knowledge regarding HIV/AIDS among the study respondents (61.6%). The 41.5% of respondents with negative attitudes may manifest some level of discrimination against PLHIV. Therefore, many health promotion activities and interventions are still needed in the Secondi-Takoradi metropolis to improve students’ attitudes towards PLHIV.

It was found in the current study that 26.2% of the respondents had a history of sexual intercourse, with 51.9% using a condom during the first sexual intercourse and 41.5% regularly using it. More than half (56.1%) of the sexually active respondents had multiple sex partners in the previous year. Some of the respondents (6.4%) were found to have used or were using injection drug equipment. These results differ from those of a cross-sectional study by Christane et al.^19^ in Libreville, Gabon, which reported that 73.0% of school students had a history of sexual intercourse, 29.6% were using condoms during sex and 25.6% used condoms regularly during sexual intercourse with casual partners.^19^ The current results also show that more students used condoms than reported by Adeleke et al.^5^ in their cross-sectional study conducted among school-going adolescents in Atisbo Local Government Area, Nigeria (20.4%). This is a cause for great concern in a population where several combined risk factors are found, but the use of condoms is still low and even inconsistent. Indeed, half of our study population did not use a condom both during their first sexual intercourse and during their last sexual encounter; however, slightly more than half of them had multiple sex partners at the time. An education on condom use is warranted.

On the other hand, a cross-sectional survey conducted among male high school students in Lao People’s Democratic Republic reported a higher figure of 31.3% of students having a history of sexual intercourse, and 70.2% of these had used a condom, compared to the current result of 20.8% and 47.4%, respectively.^31^ These discrepancies could be a result of their study involving only males, who mostly engage in risky life behaviours, as compared with the current study, which included both males and females. Here, we found inconsistent condom use; only half of the respondents used condoms during their sexual encounters, while close to 50% had unprotected sexual intercourse. This shows the discordance between the high risks of contracting HIV among these youths consistent with studies carried out elsewhere.^32^

There was association of KAPs with some socio-demographic characteristics, unlike in a study conducted in the Ejura-Sekyedumase district of Ghana, in which no association was found between the demographic characteristics and knowledge of HIV/AIDS.^10^ In our case, respondents aged 15–19 years, single, belonging to the Muslim religion and studying at school F were associated with poor knowledge on HIV/AIDS. In Congo, the DRC Demographic Health Survey has shown a relationship between ages 15 and 24 years and being unable to know that healthy people can be HIV-positive.^33^ The low level of knowledge associated with being Muslim in the current study calls for a collaborative effort between health authorities and religious leaders to address some misbeliefs among the general public. The same age range and the single marital status also were associated significantly with a poor attitude towards HIV/AIDS and HIV-positive people. This poor attitude can increase the rejection of AIDS patients and compromise the fight against HIV, such as use of condoms among youth, among others.

### Limitations

This study was restricted to only three schools in the western region of Ghana and all the participating institutions were boarding schools excluding form (grade level) three. This limits the generalisability of the current findings to other regions and today’s SHSs. The study only concentrated on knowledge as the primary factor in explaining HIV transmission among young people, which may not be so. The study failed to explore relevant points related to sexual behaviour in youths, such as some attitudes surrounding condom use, the age of sexual debut and high personal HIV risk perception. Lack of such information may impede the design of a culturally tailored message to this group able to reverse their possible bad risk behaviour. Finally, because the questionnaire was self-administered, social desirability bias may have occurred. However, the anonymity of the respondents hopefully encouraged students to be honest in their responses. Despite all of these limitations, the researchers believe that this study might be a reasonable source of information for other researchers and policymakers.

### Recommendations

Based on the findings of this study, the following suggestions can be formulated:

- Human immunodeficiency virus/acquired immunodeficiency syndrome awareness campaigns among SHS students in the metropolis should generally pay much attention to specific issues, especially regarding the transmission and management of HIV/AIDS.
- Sexuality education in schools should be reinforced to correct the misconceptions observed in the current study and encourage safe practices and positive attitudes towards PLHIV.
- Furthermore, HIV/AIDS prevention programmes must move beyond education into encouraging and enhancing voluntary counselling and testing services among students.
- Human immunodeficiency virus/acquired immunodeficiency syndrome awareness and prevention campaigns must also be targeted at young people, especially those below 20 years of age, to help improve their attitudes towards PLHIV.

Future research involving regional and national representative samples of school-attending and out-of-school adolescents could contribute substantially to HIV/AIDS prevention.

## Conclusion

The study revealed that SHS students in the three schools in which the study was conducted generally had some knowledge of the basics of HIV/AIDS, though it can be considered inadequate. However, there are still some gaps regarding the modes of transmission, such as transmission of HIV through handshake, sharing of clothes with an HIV-positive person, mosquito bite, witchcraft, etc., which need further attention. Furthermore, the knowledge and awareness on HIV/AIDS-related information did not translate to students’ decision to undergo voluntary testing, with more than four-fifths of the students not knowing their HIV status. The attitudes of respondents towards PLHIV were found to be encouraging as a slight majority had a good attitude. The majority of the respondents engaged in risky practices regarding HIV. There was no significant association between KAPs regarding HIV/AIDS and socio-demographic variables except between age and attitudes.
